# A Steady Cause of Unsteadiness: A Case of Thyroid-Associated Cerebellar Ataxia

**DOI:** 10.7759/cureus.8080

**Published:** 2020-05-12

**Authors:** Muhammad Talha Ayub, Tabinda Shafiq, Tooba Ayub, Sana Afroz, Hasan Husni

**Affiliations:** 1 Cardiovascular Medicine, Rush University Medical Center, Chicago, USA; 2 Endocrinology, University of Alabama at Birmingham, Birmingham, USA; 3 Internal Medicine, King Edward Medical University, Lahore, PAK; 4 Rheumatology, Rush University Medical Center, Chicago, USA; 5 Endocrinology, Johns Hopkins Hospital, Baltimore, USA

**Keywords:** encephalopathy with autoimmune thyroid disease, hashimoto's thyroiditis, grave's disease, thyroid peroxidase antibodies

## Abstract

Encephalopathy with autoimmune thyroid disease (EAATD) is mostly associated with Hashimoto’s thyroiditis and has been uncommonly reported with Grave’s disease. This case is aimed to report the association of EAATD with thyroid peroxidase (TPO) and thyroid-stimulating immunoglobulin (TSI) antibodies in Grave’s disease. We report a 55-year old male who presented with thyrotoxicosis and cerebellar ataxia and was diagnosed with Grave’s disease based on clinical and biochemical findings. The patient was managed with anti-thyroid medications with resolution of both thyrotoxicosis and cerebellar symptoms proving the hypothesis that patient’s encephalopathy was autoimmune and related to his thyroid disease. High index of suspicion should be maintained for EAATD in patients presenting with neurological deficits with associated clinical and biochemical evidence of autoimmune thyroid disease.

## Introduction

Encephalopathy with autoimmune thyroid disease (EAATD) is a well-known entity. It is mostly associated with Hashimoto’s thyroiditis (HT) and has been uncommonly reported with Grave’s disease (GD) [1]. Clinical presentation is variable with a relapsing and remitting course and responsiveness to the corticosteroid treatment. Patients can present with seizures, stroke-like episodes, cognitive decline, neuropsychiatric symptoms and myoclonus [1-3]. Diagnosis is suggested by high levels of anti-thyroid antibodies, increased cerebrospinal fluid (CSF) protein concentration and non-specific electroencephalogram (EEG) abnormalities [4]. The pathophysiological mechanisms underlying EAATD are not well understood. This case is aimed to report the association of EAATD with thyroid peroxidase (TPO) and thyroid-stimulating immunoglobulin (TSI) antibodies in GD. We suggest that high index of suspicion should be maintained for EAATD in patients presenting with neurological deficits with associated clinical and biochemical evidence of autoimmune thyroid disease.

## Case presentation

A 54-year-old man with past medical history of albinism and hypertension presented with progressively worsening palpitations, generalized weakness and gait unsteadiness for 10 months. He also endorsed subjective fevers, dizziness and unintentional weight loss of 45 lbs, but denied diplopia, dysphagia, syncope, urinary or bowel accidents, paresthesia and/or sensory deficits. He had no recent travel and denied any alcohol use. Family history was positive for a son with albinism. His vitals on presentation are as follows: heart rate 104/min, respiratory rate 19/min, afebrile and oxygen saturation of 97% on room air. Physical examination was remarkable for hand tremors, diffuse non-tender goiter, dysarthria, bilateral horizontal nystagmus, ataxic wide-based gait, dysdiadochokinesia and 3+ bilateral knee reflexes with intact sensations.

**Figure 1:**
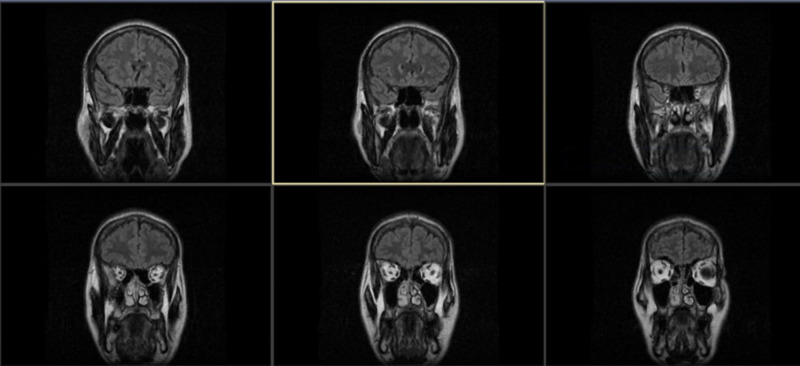
Brain MRI with and without contrast

Clinical presentation was consistent with a cerebellar syndrome with presumed etiologies as paraneoplastic, autoimmune, post-viral or degenerative ataxia. Labs showed normal complete blood count (CBC), complete metabolic panel (CMP), rapid plasma reagin (RPR), vitamin E, B12, lactate, pyruvate and anti-gliadin antibodies. Thyroid profile showed thyroid-stimulating hormone (TSH) <0.015 uIU/ml, T4 3. 61 ng/dl, TPO antibody 104 IU/ml (normal<9 IU/ml) and TSI antibody 293 IU/ml (normal<140 IU/ml). Thyroid ultrasound showed increased vascularity. CT of the head was unremarkable for any intracranial pathology. MRI of the brain did not show hyperintense T2 signals or enhancement on post-gadolinium (Gd) T1-weighted images (Figure 1). 

**Figure 2:**
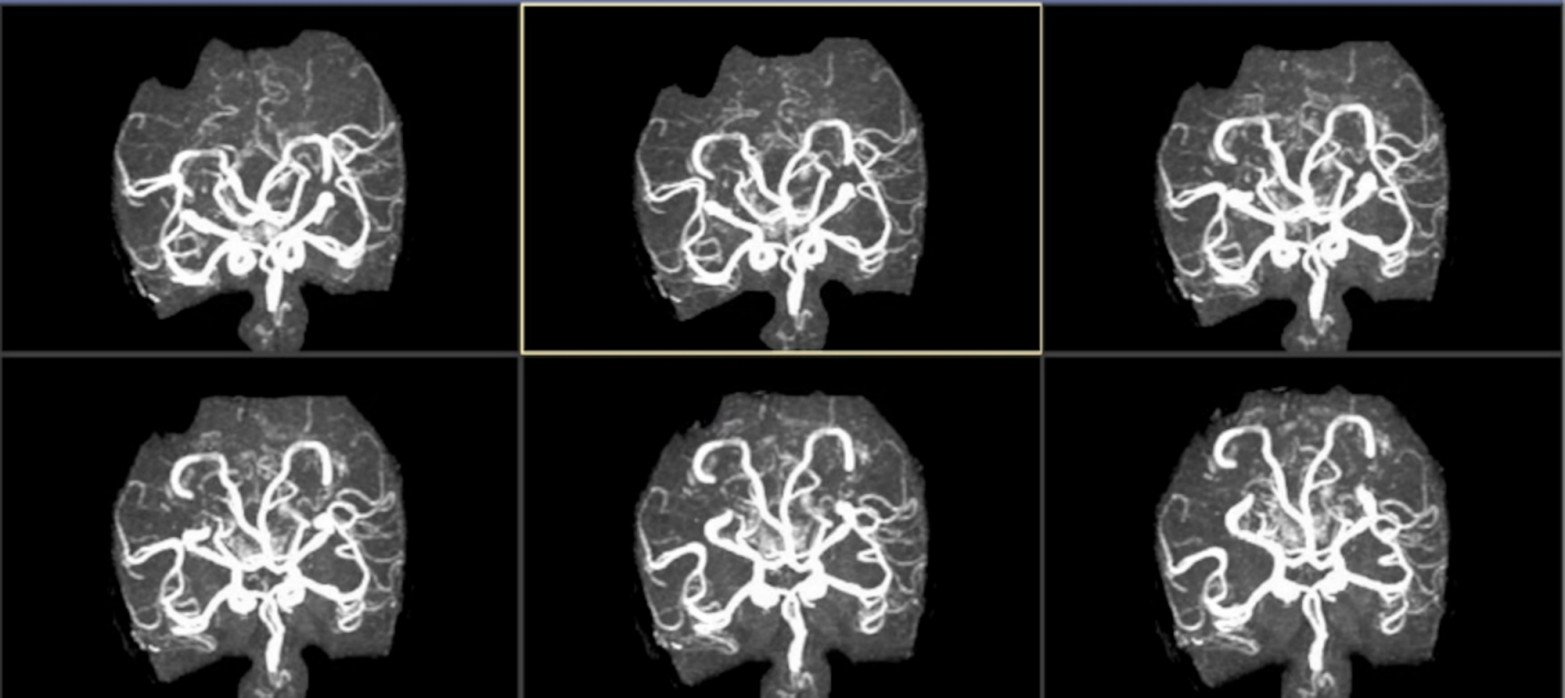
Magnetic resonance angiography of the brain

Magnetic resonance angiography of the brain did not show any findings consistent with intracerebral vasculitic process (Figure 2).

CSF analysis was remarkable for normal protein and cell counts, negative paraneoplastic antibody panel and oligoclonal bands. CSF fungal and mycobacterial cultures showed no growth. 

Diagnosis of GD was made based on clinical and biochemical evidence of thyrotoxicosis in the setting of TPO and TSI antibodies. The patient responded to metoprolol and methimazole, with improvement in tremors. Given the negative workup for structural, metabolic, infectious and vascular or paraneoplastic etiologies of cerebellar dysfunction, symptoms were attributed to autoimmune brain disease associated with GD. Definite treatment with radioactive iodine ablation therapy resulted in clinical and biochemical resolution of hyperthyroidism. The patient has demonstrated complete recovery of cerebellar signs and symptoms on subsequent outpatient follow-up. 

## Discussion

EAATD is a rare albeit important entity. It is mostly documented in association with HT [1-3]. Limited data are available for encephalopathy associated with GD [4]. Encephalopathy associated with thyroiditis or anti-thyroid antibodies is very uncommon, with an estimated prevalence of 2.1 per 100,000 habitants [5]. It occurs more commonly in females (4:1 ratio) and, although there are cases reported from childhood through the eighth decade of life, the mean age of onset is in the fourth decade [2,3,6]. The pathophysiology is not yet well defined, but there is a concomitant finding of high serum antibody levels and CSF protein [4].

Since the initial description of Hashimoto’s encephalopathy in 1966, EAATD has been described in association with different clinical presentations and has a wide clinical spectrum [2]. Cognitive and behavioral abnormalities, altered consciousness, involuntary movements (including tremors and myoclonus), seizures, epilepsy like symptoms, focal neurological signs, symptoms of encephalitis (such as headache and nausea) and ataxia are the reported clinical manifestations of EAATD [1,4,6]. 

We describe cerebellar ataxia as a feature of EAATD associated with hyperthyroid phase of GD. It is similar in presentation to HT-associated cerebellar ataxia and clinically mimics spinocerebellar degeneration. It is characterized by the absence of nystagmus, absent or mild cerebellar atrophy and lazy background activity on EEG [4].

EAATD is a diagnosis of exclusion and is diagnosed mainly by ruling out other possible causes of encephalopathy by neuroimaging and CSF analysis. Common differential diagnoses include metabolic, toxic and infectious causes of encephalopathy. Neuroimaging studies are non-revealing; however, increased level of protein in the CSF has usually been found in these cases. The nature of the autoantibodies involved in the pathogenesis of autoimmune thyroid disease and the mechanisms that cross anti-thyroid autoimmunity and encephalopathy are still undefined [7,8]. In most of the cases, the diagnosis was based on neurological symptoms, negative CSF evidence of viral or bacterial infection and presence of anti-thyroid antibodies. The hormone levels in the blood appear to be unrelated to the encephalopathy symptoms. 

Physicians’ awareness of this condition is of great importance because most patients respond dramatically to anti-thyroid therapy and show good clinical outcome as in our patient. In some cases, thyroidectomy has been proven to be helpful. This interesting report suggests that thyroid antigens may play a direct role in the pathogenesis of EAATD by triggering the immunological reaction leading to encephalopathy. The lack of differences in EAATD manifestations, findings and outcomes between patients with GD and HT suggests that the diagnosis of EAATD should be considered in all patients with signs of encephalopathy of unknown origin and an autoimmune thyroid disease, independently by the functional status of the thyroid and the nature of the underlying autoimmune thyroid disease itself.

## Conclusions

The lack of differences in EAATD manifestations, findings and outcomes between patients with GD and HT suggests that the diagnosis of EAATD should be considered in all patients with signs of encephalopathy of unknown origin and an autoimmune thyroid disease, independently by the functional status of the thyroid and the nature of the underlying autoimmune thyroid disease itself.
